# Effects of Different Types of Pectin Oligosaccharides on the Community Structure and Metabolism of Human Fecal Microbiota

**DOI:** 10.3390/microorganisms14081814

**Published:** 2026-08-17

**Authors:** Huipeng Liu, Xining Geng, Yousi Fu

**Affiliations:** 1 Department of Chemical and Biochemical Engineering, College of Chemistry and Chemical Engineering, Xiamen University, Xiamen 361005, China; m13526718841@163.com; 2 College of Chemistry and Chemical Engineering, Pingdingshan University, Pingdingshan 467000, China; geng_xn@yeah.net; 3 Research Institute of Public Health, Tianjin Key Laboratory of Food Science and Health, School of Medicine, Nankai University, No. 38, Tongyan Road, Haihe Education Park, Tianjin 300350, China

**Keywords:** pectin oligosaccharides, gut microbiota, monosaccharide composition, short-chain fatty acids, in vitro fermentation

## Abstract

Pectin oligosaccharides have emerged as promising prebiotics whose biological activities are closely tied to their structural features, particularly monosaccharide composition. However, systematic experimental evidence regarding how different monosaccharide compositions collectively influence gut microbiota modulation, SCFA production, and the metabolomic responses of mixed pectin oligosaccharides remains limited. To address this gap, Pool-I (27.92% galacturonic acid, rich in neutral sugars including galactose, glucose, and arabinose) and Pool-II (98.73% galacturonic acid) were evaluated by comparison with inulin, a reference prebiotic, using an in vitro human fecal fermentation model. Microbial community structure, SCFA production, and metabolomic profiles were assessed at 12 h and 24 h via 16S rRNA gene sequencing, gas chromatography, and untargeted metabolomics, respectively. Inulin exhibited superior enrichment of Actinobacteria (predominantly *Bifidobacterium*) and consistently higher total SCFA production (24.07 ± 0.25 mmol/L acetic acid at 24 h vs. 20.27 ± 0.28 and 20.53 ± 0.76 mmol/L for pectin oligosaccharides), particularly propionic and butyric acids. Although Pool-I and Pool-II yielded lower overall SCFA concentrations, they significantly enriched beneficial SCFA-producing genera (including *Coprococcus_3*, *Butyricicoccus*, and *Parabacteroides*) at 24 h. Metabolomic analysis revealed that Pool-I uniquely upregulated twenty-six differential metabolites (*VIP* > 1, *p* < 0.05), including taurine (*VIP* = 2.40, *p* < 0.001), L-proline (*VIP* = 1.72, *p* = 0.015), and pantothenate (*VIP* = 1.63, *p* < 0.001), while reducing uric acid (*VIP* = 1.05, *p* < 0.001), whereas only two differential metabolites were identified for Pool-II. We conclude that monosaccharide composition is a key determinant of the prebiotic efficacy of pectin oligosaccharides: Pool-II selectively promotes SCFA-producing taxa, whereas Pool-I elicits broader metabolic regulation. These effects provide a theoretical foundation for developing pectin oligosaccharides as potential prebiotic candidates.

## 1. Introduction

Gut health is critical for sustaining host general health and lowering the incidence of diseases associated with changes in nutrition and lifestyle, and its composition and function are susceptible to regulation by the fine structure of dietary fiber [1,2,3,4,5]. Pectin is a structurally complex plant polysaccharide composed of multiple domains, including homogalacturonan (HG), rhamnogalacturonan I (RG-I), and rhamnogalacturonan II (RG-II). The HG region consists of a linear backbone of galacturonic acid, while RG-I exhibits a hairy structure. Its backbone is formed by alternating rhamnose and galacturonic acid residues, with neutral sugar side chains, such as arabinose and galactose, attached [6]. In the era of precise nutrition, treating pectin as a single functional ingredient can no longer meet practical application requirements, since its biological activities are strongly dependent on structural parameters, including molecular weight, degree of esterification, and especially monosaccharide composition and side chain structure [7].

To overcome the limitations of high-molecular-weight pectin in processability and bioavailability, pectin oligosaccharides produced via controlled hydrolysis exhibit superior prebiotic potential [6,8]. Multiple studies have demonstrated that pectin oligosaccharides can promote the proliferation of *Bifidobacterium* and *Lactobacillus* in vitro, as well as enhance the growth of short-chain fatty acids [9,10]. Notably, pectin oligosaccharides with distinct fine structures (i.e., molecular weight, degree of esterification, monosaccharide composition, and glycosidic bond type) determine their fermentation rates in the gut and interactions with the gut microbiota, thereby ultimately affecting host health. For instance, Gullón et al. studied the fermentation of different oligosaccharides based on fecal microbiota and determined their utilization priority: pectin oligosaccharides > galactooligosaccharides > xylooligosaccharides > arabinose oligosaccharides > galacturonic acid oligosaccharides [11]. The results revealed that fecal microbiota from different individuals had distinct preferences for these oligosaccharides. Li et al. [12] found that oligogalacturonic acids could ameliorate obesity in mice, promote the production of exercise-derived fatty acids, and increase the abundance of *Akkermansia* and *Lactobacillus* species. The previous studies suggested the influence of structural factors such as degree of polymerization (DP), degree of esterification (DE), molecular weight (MW), branching, and methyl-esterification/saturation status [13,14], while few previous studies have specifically analyzed the regulation of intestinal microbiota by taking isolated monosaccharide composition as the key structural variable.

To address this gap, two representative pectin oligosaccharide fractions with distinct monosaccharide compositions (Pool-I and Pool-II) were selected in this study. Using an in vitro fermentation model with fecal microbiota from healthy adults, we systematically compared the effects of pectin oligosaccharides with different sugar compositions on microbial community structure and their metabolites. Crucially, these findings establish a mechanistic link between pectin oligosaccharides and gut microbial ecology, providing a much-needed theoretical anchor for the rational utilization of pectin oligosaccharides as targeted prebiotic tools.

## 2. Materials and Methods

### 2.1. Materials and Reagents

Two pectin oligosaccharides (Pool-I and II) were prepared following the methods reported in the previous study [7]. HC-C18 [Agilent 5 HCC18 (250 mm × 4.6 mm)] was obtained from Agilent Technologies (Santa Clara, CA, USA). 1-phenyl-3-methyl-5-pyrazolone (PMP), monosaccharide standards [mannose (Man), rhamnose (Rha), galacturonic acid (GalA), galactose (Gal), glucose (Glc), arabinose (Ara), and xylose (Xyl)] and short-chain fatty acid standards [acetic acid, propionic acid, and butyric acid] were purchased from Shanghai Aladdin Biochemical Technology Co., Ltd. (Shanghai, China). Other chemical reagents were of the highest available grade.

### 2.2. Monosaccharide Composition

The monosaccharide profiles of Pool-I and II were analyzed following a previously reported protocol from our research group [10]. In brief, 4 mg of each sample was hydrolyzed in 1 mL of 4 M trifluoroacetic acid (TFA) at 110 °C for 4 h under nitrogen protection. Upon cooling to ambient temperature, methanol was incorporated into the hydrolysate, and the mixture was evaporated to dryness under nitrogen. This purification step was performed in triplicate to fully eliminate residual TFA. The dried hydrolytic residue was redissolved in 100 μL of distilled water and mixed thoroughly with 0.6 M NaOH solution (100 μL). For derivatization, 50 μL of the prepared sample solution was mixed with 50 μL of 0.3 M PMP methanolic solution, and the reaction was allowed to proceed in a 70 °C water bath for 100 min. After cooling, the reaction system was neutralized using 200 μL of 0.3 M HCl solution. The neutralized solution was supplemented with methanol at an equal volume and subsequently dried under nitrogen gas. The obtained PMP derivatives were redissolved in 500 μL of distilled water and purified via triple extraction with 1 mL of chloroform. The upper aqueous layer was collected and filtered through a 0.45 μm organic membrane prior to high-performance liquid chromatography (HPLC) detection. All monosaccharide reference standards were treated and analyzed using the same method. HPLC separation was carried out using an HC-C18 column (250 mm × 4.6 mm). The chromatographic parameters were established as follows: mobile phase A consisting of 15% acetonitrile and 85% phosphate buffer (pH 6.7), and mobile phase B consisting of 40% acetonitrile and 60% phosphate buffer (pH 6.7). The detection wavelength was set at 254 nm, the column temperature was maintained at 25 °C, and the flow rate was fixed at 1 mL/min for 55 min. The gradient elution program was set as follows: 0–10 min, 100–90% phase A (0–10% phase B); 10–30 min, 90–80% phase A (10–20% phase B); 30–45 min, held at 80% phase A (20% phase B) and 45.01–55 min, returned to 100% phase A (0% phase B).

### 2.3. Collection of Feces and In Vitro Fermentation Culture

Fecal samples were obtained from three healthy donors who had been screened and qualified as donors for fecal microbiota transplantation. The collection and pretreatment procedures were based on our previous study [15], with slight modifications. Equal amounts of fecal material from the three donors were pooled to prepare a standardized gut microbiota suspension for in vitro fermentation.

In vitro fermentation was performed in 100 mL serum bottles containing 50 mL of culture medium under the clinical study registered at ClinicalTrials.gov (NCT03426683). The medium consisted of 2.0 g/L of yeast extract, 2.0 g/L of peptone, 0.1 g/L of NaCl, 0.04 g/L of KH_2_PO_4_, 0.01 g/L of MgSO_4_·7H_2_O, 0.01 g/L of CaCl_2_, 2.0 g/L of NaHCO_3_, 0.02 g/L of heme, 0.50 g/L of L-cysteine hydrochloride, 0.50 g/L of bile salts, 2 mL/L of Tween 80, and 10 µL/L of vitamin K1. The medium was flushed with nitrogen for 20 min before and after dispensing to maintain anaerobic conditions and then sterilized at 121 °C for 15 min.

After sterilization, 1 mL of a 20 mg/mL pectin oligosaccharide solution, sterilized through a 0.22 µm membrane filter, was added to the fermentation medium. Commercial inulin at the same concentration was used as a positive control, whereas 1 mL of deionized water was added to the blank control. Each treatment was performed in three parallel fermentation vessels using the same pooled gut microbiota. Samples were collected at 12 and 24 h and stored at −80 °C until further analysis.

### 2.4. DNA Extraction and PCR Amplification of Human Fecal Microbiota

The DNA extraction of human fecal microbiota was conducted using the Hipure Soil DNA extraction kit (Magen, Guangzhou, China), following the operating instructions in the manual. The PCR amplification conditions for the V3–V4 region of the 16S rRNA gene were 94 °C (2 min), 98 °C (10 s), 62 °C (30 s, 55 °C for the 16S V4 region), and 68 °C (30 s), repeated for 30 cycles. Subsequently, the primers 341F: CCTACGGGNGGCWGCAG and 806R: GGACTACHVGGGTATCTAAT were employed at a temperature of 68 °C for a duration of 5 min. PCR reactions were conducted in triplicate. Amplification system: the 50 μL mixture consists of 5 μL of 10 × KOD buffer, 5 μL of 2 mM dNTPs, 3 μL of 25 mM MgSO_4_, 1.5 μL of upstream and downstream primers (10 μM), 1 μL of KOD polymerase, and 100 ng of template DNA. The PCR-related reagents were manufactured by TOYOBO in Japan.

### 2.5. Fecal Microbiota Bioinformatics Analysis

Raw 16S rRNA gene sequencing data were processed using the QIIME pipeline (v1.9.1). Following the protocol from Fu et al. [15], the Shannon index of alpha diversity was calculated using QIIME to evaluate the richness and diversity of intestinal microbiota. Bray–Curtis distance matrices were computed for beta diversity analysis to compare community dissimilarity between groups and time points. PCoA ordination based on Bray–Curtis distances was visualized with the R vegan package (version 2.5.3). PERMANOVA with 999 permutations was used to test the statistical significance of community structural differences using the R project Vegan package (version 2.5.3).

### 2.6. Determination of Short-Chain Fatty Acids (SCFAs)

The determination of short-chain fatty acids was carried out using Agilent A7890 gas chromatography with an AT-FFAP column, 122-3232, 30 m × 0.25 mm (Agilent). Operating conditions: The initial temperature was kept at 80 °C for 3 min, then increased to 200 °C at a rate of 10 °C/min, and finally maintained at this temperature for 6 min. The detector was an FID hydrogen flame, with a hydrogen flow rate of 40 mL/min, an air flow rate of 350 mL/min, a nitrogen flow rate of 25 mL/min, a split ratio of 10:1, and a column flow rate of 2 mL/min. Sample processing method: A certain amount of the fecal sample was taken and centrifuged for 10 min (12,000 rpm). An appropriate amount of hydrochloric acid was then added to the supernatant to keep its pH below 3, and then the mixture was filtered through a 0.22 µm organic filter membrane for testing.

### 2.7. Analysis of Metabolites in Fermentation Broth

Amounts of 100 µL of fermentation broth (12 and 24 h), 400 µL of ice acetonitrile, and 400 µL of ice methanol were mixed. The mixture was vortexed for 60 s, followed by cell disruption using a cell breaker for 20 s. The solution was quickly frozen with liquid nitrogen and continuously crushed with a cell breaker for 20 s, repeating this process 20 times. The resulting mixture was maintained at −20 °C for 60 min. Afterwards, centrifugation was performed at 12,000 rpm and 4 °C for 15 min, and the supernatant was collected. After freeze-drying, the supernatant sample was submitted to the Biological Instrument Shared Platform of Xiamen University for LC-MS analysis using an AB Sciex TripleTOF 5600+ mass spectrometer (AB Sciex, Framingham, MA, USA).

The chromatographic conditions were as follows: an Acquity UPLC BEH Amide column (100 mm × 2.1 mm, 1.7 µm) was used. Mobile phase A consisted of water containing 25 mM ammonium acetate and 25 mM ammonium hydroxide, and mobile phase B was pure acetonitrile. The injection volume was 3 µL, the autosampler tray temperature was maintained at 4 °C, and the flow rate was 0.2 mL/min. The gradient elution program was as follows: 0–1 min, 5% phase A and 95% phase B; 1–14 min, 35% phase A and 65% phase B; 14–16 min, 60% phase A and 40% phase B; 16–18 min, held at 60% phase A and 40% phase B; and 18.1–23 min, 91% phase A and 9% phase B. The mass scanning range was set from *m*/*z* 60 to 1250. The initial data need to be preprocessed using software, and the identification of the substances was mainly carried out using the OSI-SMMS software database. Then, the data are imported into Metabianalysis for data analysis and processing.

To identify global differences in metabolic profiles between groups, we employed supervised orthogonal partial least squares discriminant analysis (OPLS-DA) [16]. We performed multidimensional analysis combining OPLS-DA and Student’s *t*-test, and identified metabolites with variable importance for the projection (*VIP*) > 1 and *p*-value < 0.05 as differential metabolites. Then, metabolites with significant differences (*p* < 0.05) were analyzed in the Kyoto Encyclopedia of Genes and Genomes (KEGG) databases. The screening criterion for differential metabolites was *VIP* > 1, while the significance threshold for the KEGG pathway enrichment analysis was *p* < 0.05. The correlation between the *VIP* metabolic sets of differential metabolites and intestinal microbiota after gut fermentation was analyzed at the genus level, and correlation analysis between dominant gut microbes and differential metabolites was performed based on Spearman’s correlation coefficient [1].

### 2.8. Data Analysis

Data are presented as the mean ± standard deviation (SD) of three parallel fermentation replicates performed using the same pooled gut microbiota. Two-way analysis of variance (ANOVA) followed by Tukey’s HSD post hoc test was used for multiple comparisons where appropriate using IBM SPSS 20.0 software. A *p*-value < 0.05 was considered statistically significant.

## 3. Results

### 3.1. Monosaccharide Composition Analysis of Pool-I and II

Table 1 shows the test results of the monosaccharide composition proportions of Pool-I and Pool-II pectin oligosaccharides. The proportion of neutral sugars in Pool-I is significantly higher than that of acidic sugars, while acidic sugars account for a far larger proportion than neutral sugars in Pool-II. There are obvious differences in carbohydrate components between the two pectin oligosaccharides, and their monosaccharide composition is also markedly distinct from that of inulin. Therefore, conducting fecal microbiota experiments on these three different types of oligosaccharides can better illustrate the correlation between carbohydrates and intestinal microbiota.

### 3.2. Effect of Different Pectin Oligosaccharides on Microbial Diversity

To evaluate the regulatory effects of different purified oligosaccharide fractions (Pool-I and Pool-II) on human intestinal microbiota, in vitro anaerobic fermentation was performed using human fecal microbiota as the inoculum. A commercial inulin group was set as the positive control because it is a well-established prebiotic that works by selectively enriching beneficial gut microbiota, boosting the production of SCFAs, and driving a range of metabolic, immune, and anti-inflammatory beneficial pathways [17], and is structurally maximally distinct from pectin oligosaccharides in terms of monosaccharide composition and glycosidic linkage type, thereby providing the clearest reference frame for evaluating composition-dependent effects. Microbial community alterations were analyzed at 12 h and 24 h during fermentation, and both α-diversity and overall community structure were characterized based on 16S rRNA gene sequencing.

**Figure 1:**
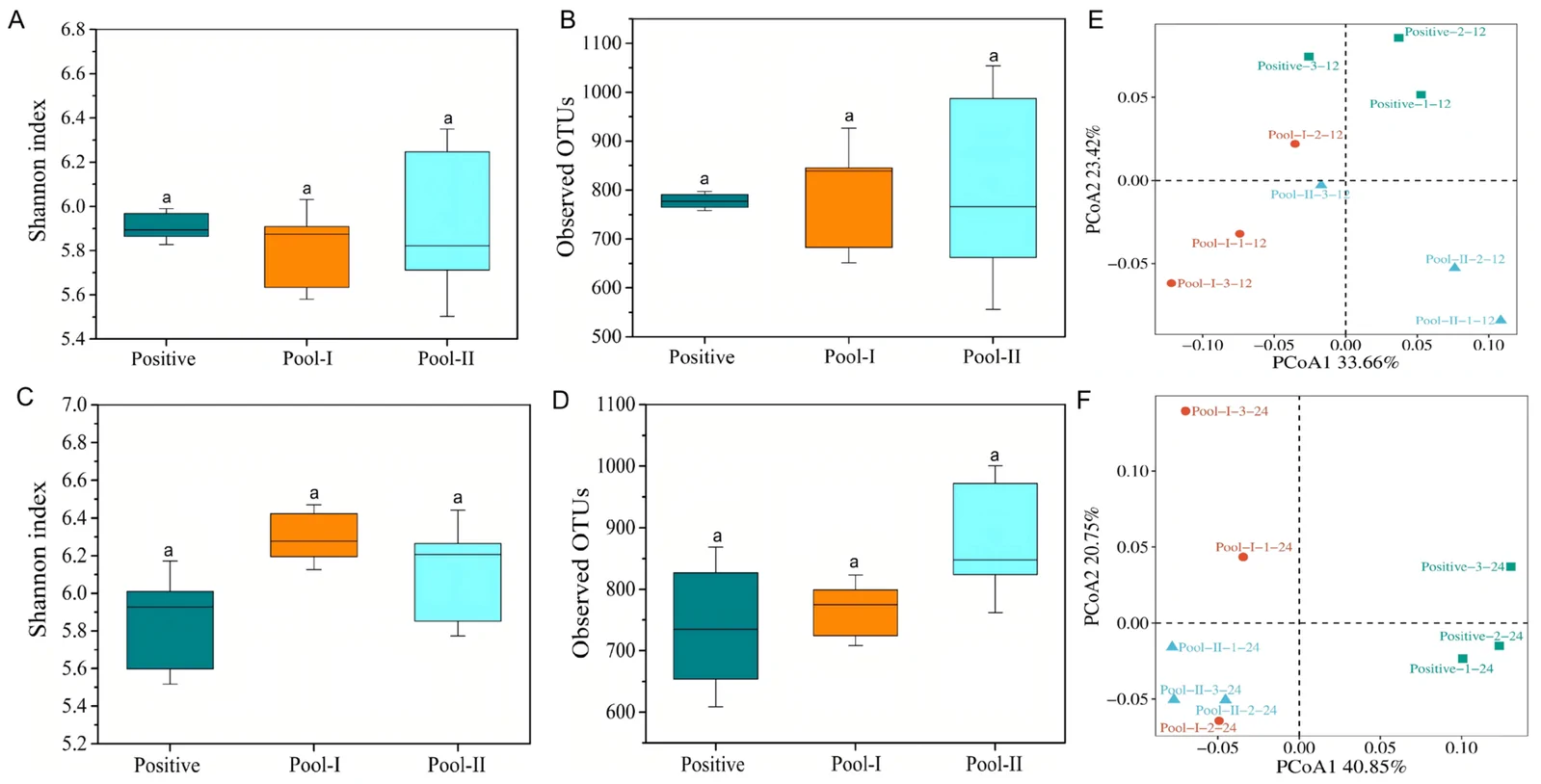
Effects of different oligosaccharides on human fecal flora: at 12 h ((**A**) Shannon index, (**B**) observed OTUs, (**E**) principal coordinate analysis (PCoA)); at 24 h (**C**) Shannon index, (**D**) observed OTUs, (**F**) principal coordinate analysis (PCoA)). Note: Data are presented as the mean ± standard deviation (*n* = 3). Different lowercase letters (a, b, and c) within the same row indicate significant differences between groups according to Tukey’s HSD post hoc test (*p* < 0.05). Panels (**E**,**F**) show principal coordinate analysis (PCoA) based on Bray–Curtis dissimilarity. PERMANOVA (999 permutations) confirmed the significant separation of microbial community composition among the three substrates at both time points (12 h: pseudo-F = 2.43, R^2^ = 0.448, *p* = 0.006; 24 h: pseudo-F = 3.27, R^2^ = 0.521, *p* = 0.006). Homogeneity of multivariate dispersions was assessed using PERMDISP (betadisper, 999 permutations), which was non-significant at 12 h (F = 0.79, *p* = 0.644) but significant at 24 h (F = 5.79, *p* = 0.033).

Microbial α-diversity was assessed using the Shannon index and observed species number, which reflect microbial community evenness and species richness [18], respectively (Figure 1A–D). All fermentation treatments resulted in significantly lower Shannon indices and observed OTU counts compared to the 0 h fecal microbiota (*p* < 0.05, Shannon: 8.55; observed OTUs: 2552), suggesting that the in vitro system generally reduced both species diversity and richness. At 12 h (Figure 1A,B), no significant differences were detected among the inulin positive control and the two oligosaccharide groups (Pool-I and Pool-II) regarding these two alpha-diversity metrics (*p* > 0.05). This pattern persisted at 24 h (Figure 1C,D), with all three groups still exhibiting no statistically significant inter-group differences (*p* > 0.05).

However, Bray–Curtis-based PCoA analyses revealed distinct community differentiation patterns at both time points (Figure 1E,F). At 12 h, PCoA1 and PCoA2 explained 33.66% and 23.42% of the total variation, respectively, while by 24 h, the first two principal coordinates cumulatively accounted for 61.60% of the structural variation. PCoA based on Bray–Curtis dissimilarity revealed clear clustering separation among the three substrate groups at both fermentation stages. PERMANOVA (999 permutations) confirmed that community composition differed significantly among the three groups at both time points (12 h: pseudo-F = 2.43, R^2^ = 0.448, *p* = 0.006; 24 h: pseudo-F = 3.27, R^2^ = 0.521, *p* = 0.006). Homogeneity of multivariate dispersions was verified using PERMDISP (betadisper), which was non-significant at 12 h (F = 0.79, *p* = 0.644) but significant at 24 h (F = 5.79, *p* = 0.033), indicating that the 24 h separation may be partly attributable to differences in within-group dispersion in addition to centroid location. Collectively, these findings indicate that although post-fermentation microbial diversity and richness were considerably lower than those of the initial fecal sample, commercial inulin, Pool-I, and Pool-II each reshaped the gut microbial community composition in a distinctive manner. Notably, the two purified oligosaccharides exhibited regulatory profiles on in vitro fermentation diversity and community structure that were clearly distinct from those of commercial inulin.

### 3.3. Effect of Different Pectin Oligosaccharides on Microbial Composition

**Figure 2:**
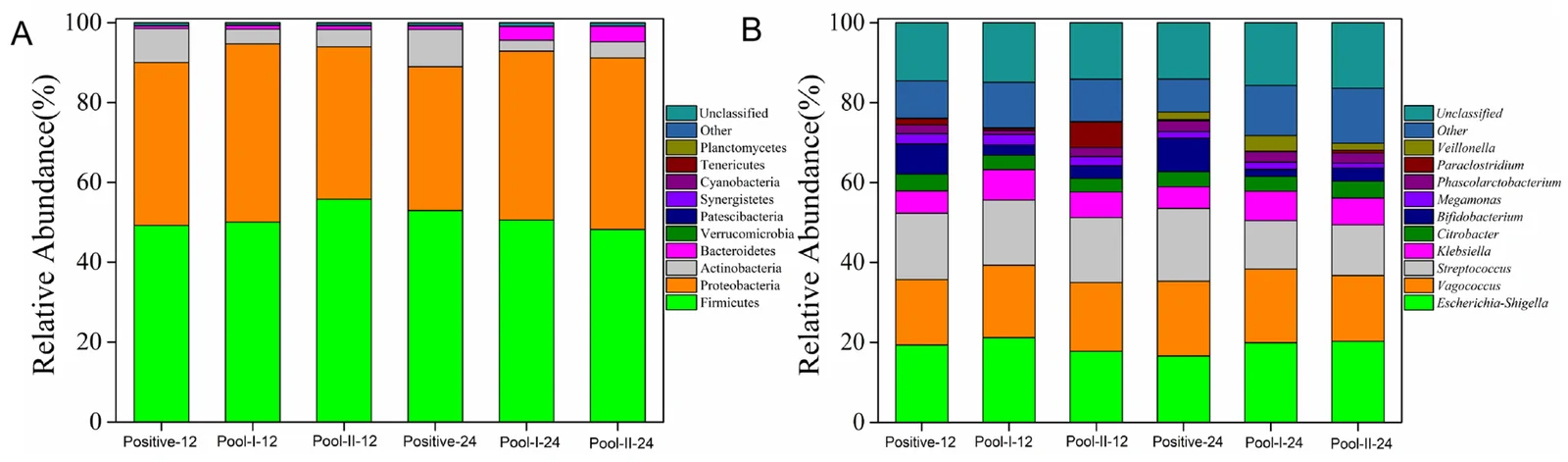
Bar chart showing the relative abundances of dominant bacteria at the phylum (**A**) and genus (**B**) levels.

To further explore the effects of different pectin oligosaccharides and inulin on microbial community composition during in vitro culture for 12 h and 24 h, we first analyzed the microbial dynamics at the phylum level. Firmicutes, Proteobacteria, Bacteroidetes, and Actinobacteria were the four dominant phyla. At the phylum level (Figure 2A and Table S1), only Actinobacteria showed significant differences among the three treatment groups at 12 h, with the inulin group (8.49 ± 0.36%) being significantly higher (*p* < 0.05) than the Pool-I (3.68 ± 1.04%), Pool-II (4.28 ± 0.41%), and blank control (4.32 ± 0.29%) groups, indicating that the enrichment of Actinobacteria was primarily attributable to the specific promoting effect of inulin. Consistent with our findings, Rui et al. [19] also reported that inulin supplementation could significantly elevate the abundance of Actinobacteriota. Firmicutes, Proteobacteria, and Bacteroidetes showed no significant differences between any treatment group and the blank control at 12 h (*p* > 0.05). Inulin consistently maintained a significantly higher Actinobacteria abundance (9.31 ± 0.54%) than Pool-I (2.71 ± 0.42%), Pool-II (4.04 ± 0.48%), and the blank control (3.10 ± 0.10%) at 24 h (*p* < 0.05), confirming inulin’s superior sustained enrichment capacity for Actinobacteria. Meanwhile, Bacteroidetes were significantly more abundant in Pool-II (3.95 ± 0.60%) and Pool-I (3.46 ± 1.30%) than in the inulin group (0.93 ± 0.16%) (*p* < 0.05), indicating that pectin-type oligosaccharides exhibit a stronger enrichment effect on the Bacteroides phylum compared to inulin.

To further explore the differential regulatory effects of two structurally distinct pectin oligosaccharide fractions (Pool-I and Pool-II) and inulin on intestinal microbial communities, the differential functional bacteria were emphatically analyzed (Table S2). At the genus level, *Escherichia-Shigella* showed significantly higher abundance in Pool-I than in the blank control at 12 h (*p* < 0.05), but no differences were observed among all groups at 24 h (*p* > 0.05); *Streptococcus* was significantly enriched by inulin at 24 h compared to Pool-I, Pool-II, and the blank control (*p* < 0.05); *Klebsiella* was significantly higher in Pool-I than in the inulin group and blank control at both time points (*p* < 0.05); *Citrobacter* was significantly higher in the blank control than in all treatment groups at both time points (*p* < 0.05), with no differences among the three treatments (*p* > 0.05); *Bifidobacterium* was consistently and significantly higher in the inulin group than in Pool-I, Pool-II, and the blank control at both time points (*p* < 0.05); *Megamonas* was significantly higher in Pool-I and the inulin group than in Pool-II and the blank control at 12 h (*p* < 0.05); *Phascolarctobacterium* was significantly higher in the blank control than in Pool-I at 12 h (*p* < 0.05); *Paraclostridium* was significantly higher in the blank control than in Pool-I and the inulin group at 12 h (*p* < 0.05); and *Veillonella* was significantly higher in Pool-I and the blank control than in Pool-II and the inulin group at 24 h (*p* < 0.05). Additionally, and importantly, *Coprococcus_3* and *Butyricicoccus*, both crucial beneficial intestinal bacteria that synthesize short-chain fatty acids [20,21,22,23], were significantly enriched by Pool-I and Pool-II (for *Coprococcus_3*) or by Pool-I alone (for *Butyricicoccus*) at 24 h compared to the inulin group and/or blank control (*p* < 0.05).

### 3.4. Effects of Different Pectin Oligosaccharides on Fermentation In Vitro on SCFAs

**Figure 3:**
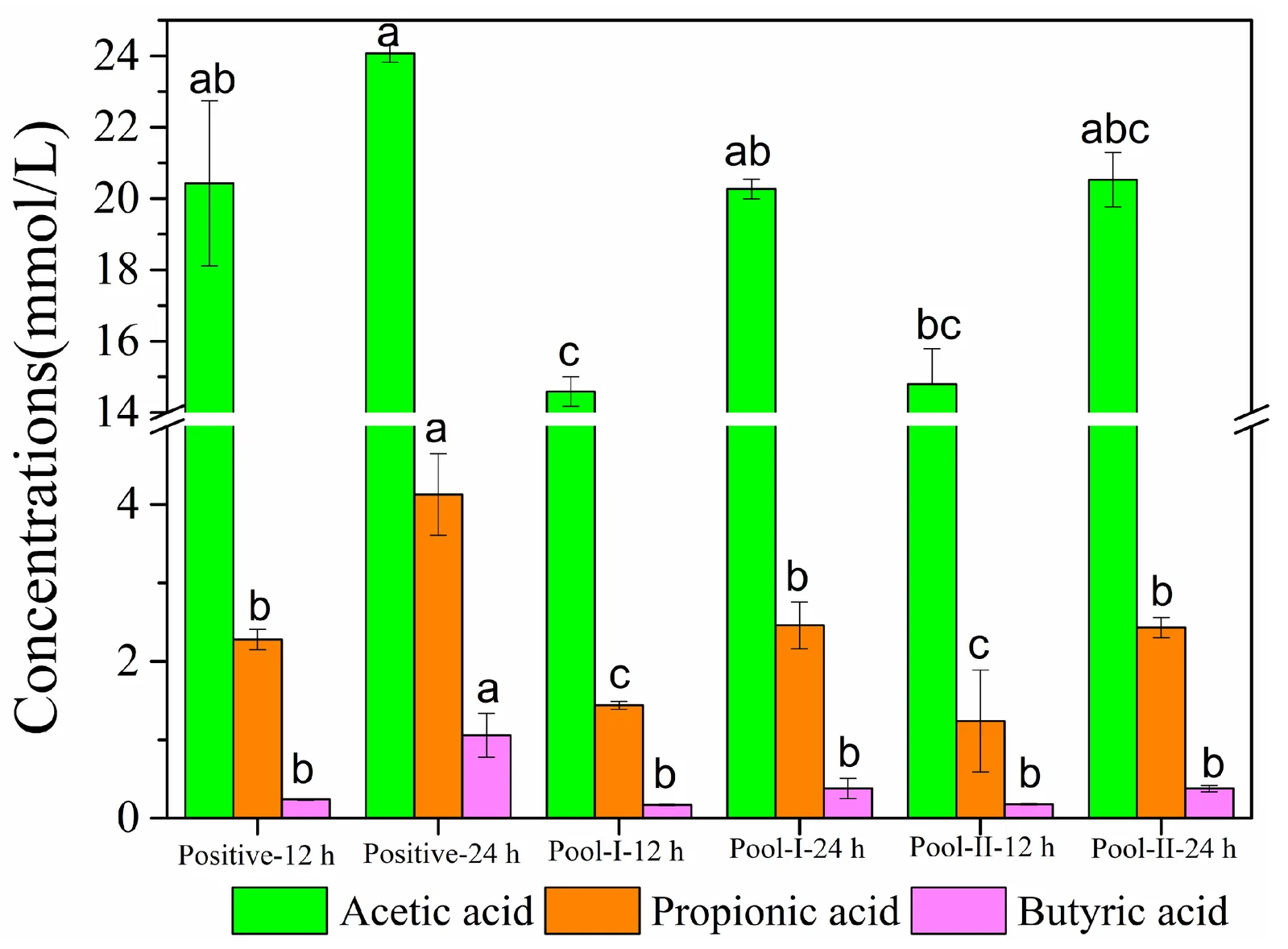
Short-chain fatty acid concentrations in in vitro fecal fermentation with different oligosaccharides (mmol/L). Note: Data are presented as the mean ± standard deviation (*n* = 3). Different lowercase letters (a, b, and c) within the same row indicate significant differences between groups according to Tukey’s HSD post hoc test (*p* < 0.05).

Short-chain fatty acids (SCFAs) are important indicators for evaluating the fermentation activity of gut microbiota and their potential health benefits [24]. The production profiles of SCFAs (including acetic, propionic, and butyric acids) across groups are presented in Figure 3. At 12 h, the concentrations of acetic acid, propionic acid, and butyric acid in the inulin group (20.43 ± 2.32, 2.28 ± 0.13, and 0.24 ± 0.01 mmol/L, respectively) were higher than those in Pool-I (14.59 ± 0.41, 1.44 ± 0.05, and 0.17 ± 0.01 mmol/L) and Pool-II (14.80 ± 0.99, 1.24 ± 0.65, and 0.18 ± 0.01 mmol/L), with propionic acid being significantly higher than in both pectin oligosaccharide groups and acetic acid being significantly higher than in Pool-I (*p* < 0.05), indicating that inulin exhibited a superior rate of SCFA production over the two pectin oligosaccharides in the early fermentation stage. At 24 h, the concentrations of all three SCFAs increased across all groups. The inulin group maintained the highest levels of acetic acid (24.07 ± 0.25 mmol/L), propionic acid (4.13 ± 0.52 mmol/L), and butyric acid (1.06 ± 0.28 mmol/L). However, the acetic acid concentrations in Pool-I (20.27 ± 0.28 mmol/L) and Pool-II (20.53 ± 0.76 mmol/L) approached those of the inulin group with no significant differences, while propionic acid and butyric acid remained significantly lower than those in the inulin group (*p* < 0.05). Notably, SCFA production increased with fermentation time across all groups, with butyric acid showing the most pronounced increase (from 0.24 to 1.06 mmol/L in the inulin group, representing an approximately 4.4-fold increase), indicating that all three substrates could be effectively utilized by gut microbiota for sustained acid production. Inulin supplementation yielded higher SCFA production than all pectin oligosaccharide groups, which could be attributed to its strong enrichment effect on *Bifidobacterium*. The relative abundance of *Bifidobacterium* in the inulin group was nearly twice that of the pectin oligosaccharide groups, and this genus is primarily responsible for acetic acid synthesis [25]. Additionally, inulin significantly promoted butyric acid accumulation compared with all pectin oligosaccharide treatments at 24 h, which was consistent with previous findings reported by Ji et al. [26].

### 3.5. Metabolomic Analysis of Human Fecal Microbiota

**Figure 4:**
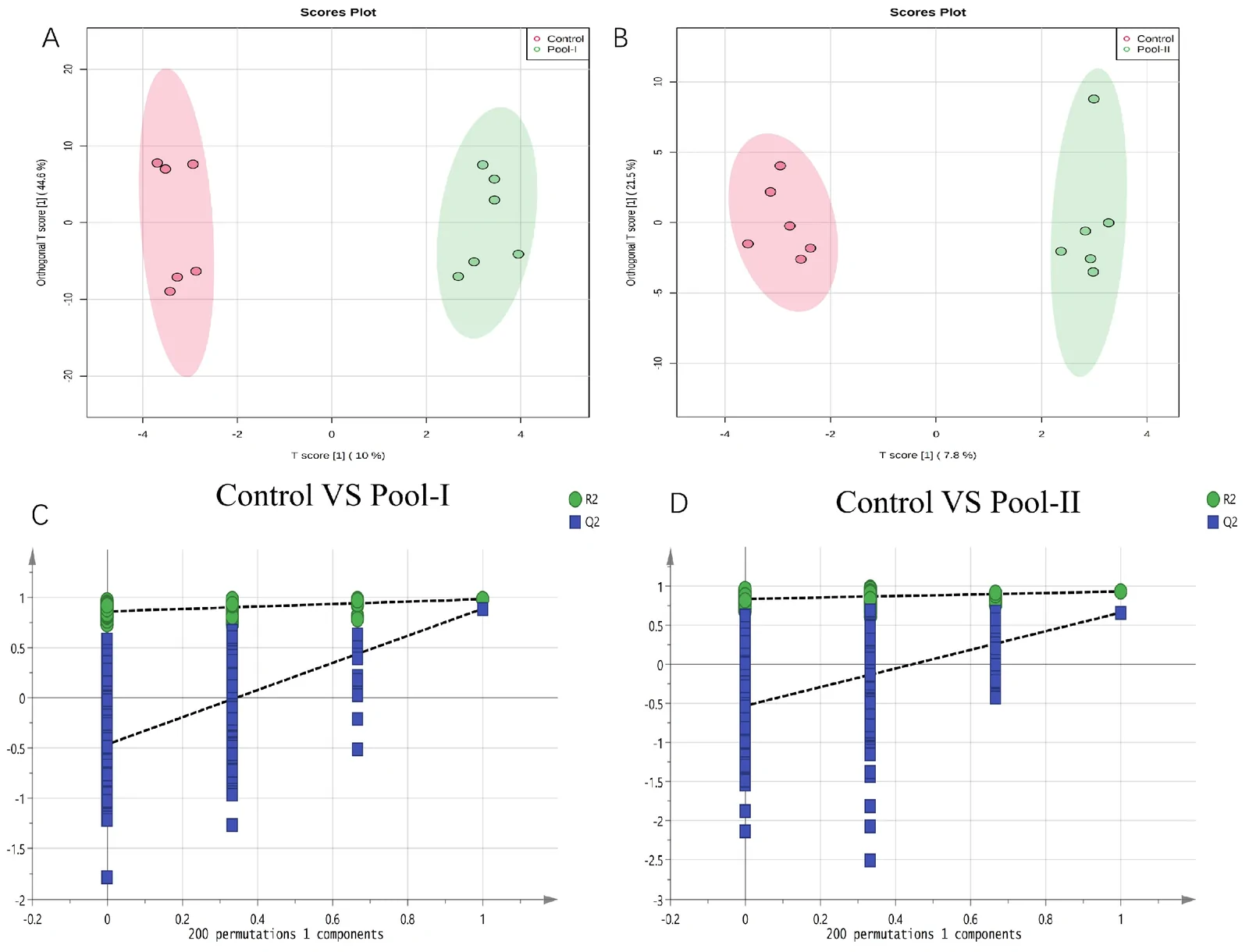
OPLS-DA score plots of fecal metabolite profiles ((**A**): control vs. Pool-I; (**B**): control vs. Pool-II) and the corresponding permutation tests (*n* = 200) for model validation ((**C**): control vs. Pool-I; (**D**): control vs. Pool-II).

Metabolites derived from gut microbiota supply energy for intestinal epithelial cells and modulate downstream cellular signaling cascades, serving as a critical functional bridge linking host health and gut microbial homeostasis [27]. Lamichhane et al. [28] demonstrated that gut microbial dysbiosis is not the sole contributor to altered host health status; instead, microbiota-derived metabolites exert more extensive systemic regulatory effects, particularly in modulating immune function. Metabolic profiling data in this study were primarily obtained via ultra-high-performance liquid chromatography coupled with mass spectrometry (UPLC-MS), with all testing procedures completed at the Biomedical Instrument Reservation Platform of Xiamen University. The orthogonal partial least squares discriminant analysis (OPLS-DA) model in Figure 4A,B illustrates the metabolic differences between groups supplemented with two types of pectin oligosaccharides and the blank control group. The model results revealed distinct metabolic profiles in the two pectin oligosaccharide treatment groups relative to the blank control. The permutation displacement test (Figure 4C,D) further verified that the established OPLS-DA models efficiently distinguished inter-group metabolic differences, enabling accurate screening of differential metabolites among groups. Differential metabolites with variable importance in projection (*VIP*) > 1 and *p* < 0.05 were screened and summarized in Table 2. Following annotation against the Human Metabolome Database (HMBD), a total of 26 differential metabolites were identified in the control vs. Pool-I comparison. Specifically, Pool-I supplementation significantly upregulated the levels of taurine, L-proline, pantothenic acid, D-threo-isocitrate, citrate, methionine sulfoxide, 2-hydroxy-4-methylvaleric acid, 2-hydroxyhexanoic acid, 2-ethyl-2-hydroxybutyric acid, malic acid, and hypoxanthine. In addition, fewer differential metabolites were identified in the other treatment groups (two differential metabolites in Pool-II). For the Pool-II group, 3-aminobutyric acid was significantly upregulated while guanine was downregulated.

**Figure 5:**
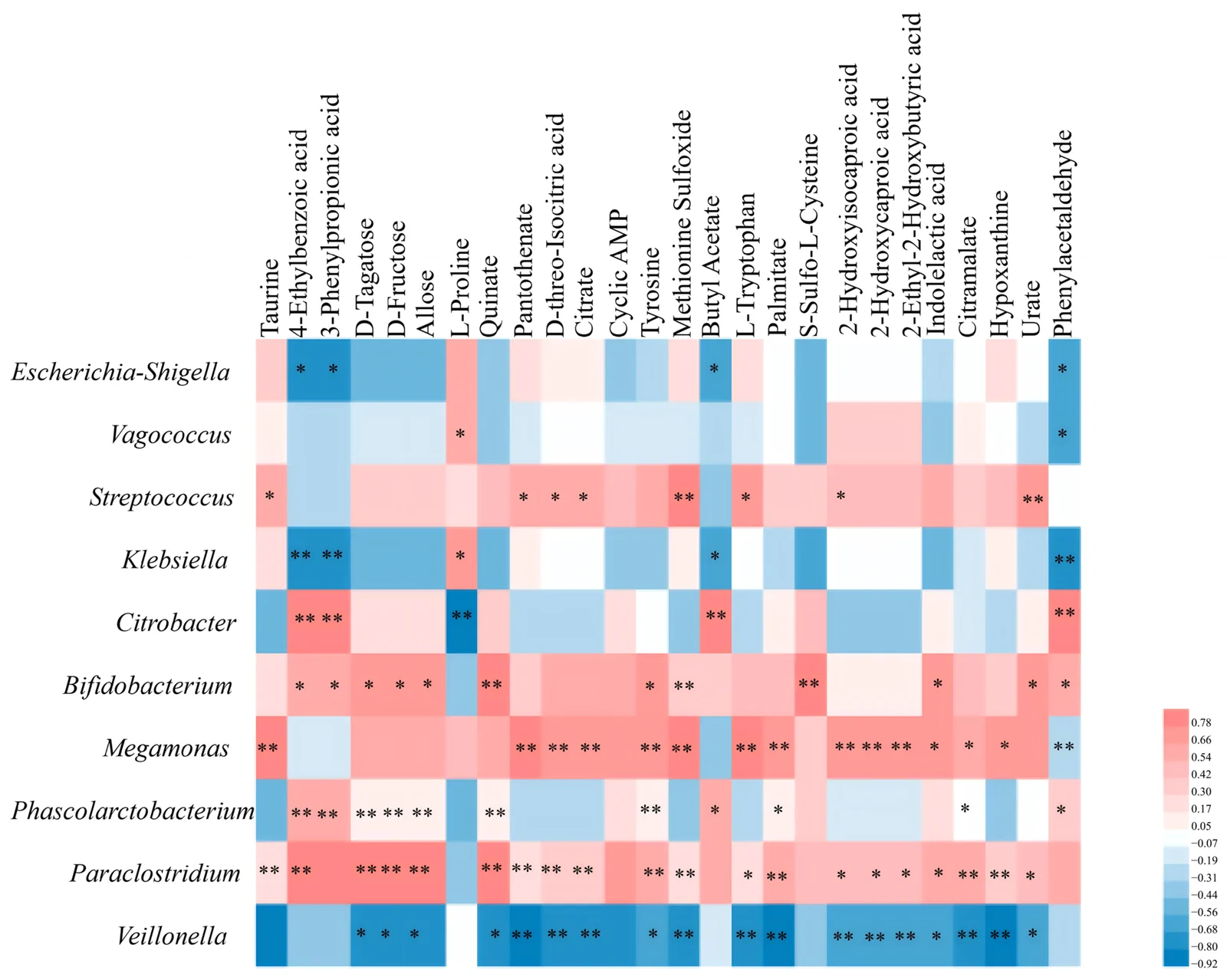
Correlation analysis between differential metabolites and fecal flora (genus level): control vs. Pool-I, * *p* < 0.05, ** *p* < 0.01.

Metabolic pathway enrichment analysis showed that the differential metabolites identified in the control vs. Pool-I comparison were significantly enriched in the phenylalanine metabolism pathway (*p* = 0.0125). The limited number of differential metabolites in the Pool-II group precluded further pathway enrichment analysis. Figure 5 presents the correlation analysis between differential metabolites and fecal microbiota at the genus level. The results demonstrated that the abundance fluctuations of *Bifidobacterium*, *Megamonas*, *Phascolarctobacterium*, *Paraclostridium*, and *Veillonella* were strongly correlated with the variations in differential metabolite levels (*p* < 0.05). Specifically, *Veillonella* abundance was negatively correlated with the content of differential metabolites, while the abundances of *Bifidobacterium*, *Megamonas*, *Phascolarctobacterium*, and *Paraclostridium* showed predominantly positive correlations with the accumulated differential metabolites (*p* < 0.05).

## 4. Discussion

This study systematically compared the regulatory effects of two pectic oligosaccharide fractions with distinctly different monosaccharide compositions and inulin on human fecal microbiota and their metabolites. The results revealed that all three substrates could effectively remodel the gut microbiota structure and stimulate the production of SCFAs, yet their regulatory patterns differed significantly. Pool-II consists almost entirely of galacturonic acid, making it a homogalacturonan oligosaccharide (Table 1). The relatively uniform monosaccharide composition may account for its selective enrichment of Bacteroidetes at 24 h (Table S1), as members of the phylum Bacteroidetes are known to possess extensive polysaccharide utilization loci and are particularly adapted to pectin-derived substrates [29]. In contrast, Pool-I consists of a complex mixture of neutral sugars, namely galactose, glucose, and arabinose (Table 1). It can supply a wider range of fermentable saccharides and potentially sustain more diverse metabolic activities. Nevertheless, compared with inulin—a well-established prebiotic substrate predominantly made up of fructose units connected via β-(2 → 1) glycosidic linkages—the two pectic oligosaccharide fractions yielded lower total SCFA concentrations, especially propionic and butyric acids [30]. The fructan backbone of inulin is preferentially utilized by *Bifidobacterium*, which serves as a key contributor to acetate and butyrate biosynthesis [17]. That finding is consistent with those in the present study (Table S2). It should be noted that the relative abundances of *Escherichia-Shigella* and *Klebsiella* were higher in the Pool-I group than in the blank control (Table S2). One possible reason for this is that the structural characteristics of the substrate may be influencing the outcome. In a comparable in vitro fermentation study of structurally distinct sugar beet polysaccharide fractions, the relative abundance of *Escherichia-Shigella* ranged widely across fractions, instead appearing more closely related to specific structural characteristics such as the degree of methylesterification and the relative proportions of the RG-I and RG-II domains [31]. Since the *Escherichia-Shigella* group and *Klebsiella* include opportunistic pathobionts, their potential enrichment should be taken into consideration in the safety assessment of Pool-I, and verification using species-specific quantitative PCR and culture-based methods will be required in future work.

The two pectic oligosaccharide fractions exhibited prominent advantages in enriching other SCFA-producing microbiota. At 24 h, both Pool-I and Pool-II markedly elevated the relative abundances of *Coprococcus_3* and *Butyricicoccus*, two critical butyrate-producing genera, compared with the inulin and blank control groups (Table S2). Differences in SCFA profiles further underline the functional significance of substrate structures. Although inulin consistently yielded the highest concentrations of acetate, propionate, and butyrate, Pool-I and Pool-II still produced considerable amounts of SCFAs, with their concentrations rising substantially from 12 h to 24 h (Figure 3). Notably, the acetic acid concentrations of Pool-I and Pool-II at 24 h were comparable to those of the inulin group without significant differences, demonstrating that both pectic oligosaccharide pools could sustain acetic acid production equivalent to that of inulin at the late fermentation stage (Figure 3). Nevertheless, the concentrations of propionic and butyric acids in the pectic oligosaccharide groups remained markedly lower than those in the inulin group (Figure 3). The lower propionic and butyric acid levels observed in Pool-I and Pool-II may reflect their weaker enrichment efficiency for these specific propionate- and butyrate-producing taxa compared with inulin, despite the significant enrichment of *Coprococcus_3* and *Butyricicoccus* induced by the two pectic oligosaccharide fractions (Figure 3 and Table S2).

Metabolomic analysis further uncovered the unique capacity of Pool-I in altering the concentrations of fermentation-derived metabolites with potential biological relevance. Supplementation with Pool-I significantly increased the fermentation-derived metabolites of taurine, L-proline, and pantothenic acid (Table 2). Taurine plays vital roles in bile acid conjugation, antioxidant defense, and cardio protection [32]. Proline serves as an essential substrate for mucin synthesis and intestinal epithelial repair, and dietary proline supplementation has been proven to improve intestinal health in rats with DSS-induced colitis [33]. Pantothenic acid (vitamin B5), one of the thirteen essential vitamins for humans, participates in core energy metabolism pathways, facilitates the catabolism of fats and proteins, regulates blood cholesterol levels, maintains nervous system stability, and modulates the synthesis of erythrocytes and multiple hormones. D-threo-isocitrate, citrate, 2-hydroxy-4-methylvaleric acid, 2-hydroxyhexanoic acid, 2-ethyl-2-hydroxybutyric acid, and malic acid are typical organic acids that can reduce intestinal pH. This acidic microenvironment effectively inhibits the growth of intestinal pathogens such as *Escherichia coli*, optimizes gut microbial community structure, and enhances host immune function [34]. Moreover, our results showed that Pool-I pectin oligosaccharide supplementation reduced the fermentation-derived metabolites of amino acids, organic acids, and uric acid compared with the blank control. Correlation analysis between differential metabolites and fecal microbiota at the genus level revealed that the abundances of *Bifidobacterium*, *Megamonas*, *Phascolarctobacterium*, *Paraclostridium*, and *Veillonella* are strongly correlated with fermentation-derived metabolites. Notably, the positive correlations between *Bifidobacterium* and multiple beneficial metabolites further support its central role in mediating substrate-induced metabolic benefits.

In summary, the present study demonstrated that pectic oligosaccharides with distinct monosaccharide compositions exert differential modulatory effects on the intestinal microbial community and its metabolites. Pool-II efficiently enriched Bacteroidetes and SCFA-producing genera owing to its high galacturonic acid content, while Pool-I elicited broader metabolomic effects attributed to its complex neutral sugar profile. These findings provide valuable insights into the structure–function relationship of pectic oligosaccharides.

## 5. Conclusions

This study systematically compared the regulatory effects of two structurally distinct pectin oligosaccharide fractions (Pool-I and Pool-II) with the established prebiotic inulin on human fecal microbiota composition, SCFA production, and metabolomic profiles using an in vitro fermentation model. Our findings demonstrate that monosaccharide composition is a key determinant of the prebiotic efficacy of pectin oligosaccharides. Inulin exhibited the strongest overall SCFA production and preferentially enriched *Bifidobacterium*, confirming its well-established prebiotic efficacy. The pectin oligosaccharide fractions did not match inulin in total SCFA output, with propionate and butyrate concentrations remaining significantly lower than those of inulin throughout fermentation. Nevertheless, the two fractions distinctly and significantly enriched a different set of beneficial taxa that were not comparably enriched by inulin, including the butyrate-producing genera *Coprococcus_3* and *Butyricicoccus*, and *Parabacteroides* species. Moreover, Pool-II sustained acetate production equivalent to that of inulin at the late fermentation stage. Pool-I additionally upregulated multiple health-relevant metabolites such as taurine, L-proline, and pantothenate while reducing uric acid levels under in vitro conditions. Additional investigations using in vivo models and dose–response study designs are required to confirm these results and further clarify their potential implications. Follow-up research should also validate the physiological efficacy via intestinal epithelial cell models, animal trials, or human intervention studies.

## Figures and Tables

**Table 1:** Analysis of the monosaccharide composition of Pool-I and II.

Samples	Monosaccharides (%)						
	Mannose	Rhamnose	Galacturonic Acid	Galactose	Glucose	Arabinose	Xylose
Pool-I	0.11	1.01	27.92	33.20	26.01	10.27	1.25
Pool-II	0.09	0.16	98.73	0.09	0.37	0.10	0.11

**Table 2:** The different metabolites of human fecal flora in different pectin oligosaccharide groups.

Metabolites	*VIP*	*p*	Changes
Control vs. Pool-I			
Phenylacetaldehyde	2.42	0.024	lower
Taurine	2.40	<0.001	higher
4-Ethylbenzoic Acid	2.14	0.005	lower
3-Phenylpropionic Acid	2.14	0.005	lower
D-Tagatose	1.89	0.001	lower
D-Fructose	1.89	0.001	lower
Allose	1.89	0.001	lower
L-Proline	1.72	0.015	higher
Quinate	1.65	<0.001	lower
Pantothenate	1.63	<0.001	higher
D-threo-Isocitric Acid	1.55	0.003	higher
Citrate	1.55	0.003	higher
Cyclic AMP	1.38	0.006	lower
Tyrosine	1.37	<0.001	lower
Methionine Sulfoxide	1.33	<0.001	higher
Butyl Acetate	1.29	0.029	lower
L-Tryptophan	1.28	<0.001	lower
Palmitate	1.25	<0.001	lower
S-Sulfo-L-Cysteine	1.33	<0.001	lower
2-Hydroxyisocaproic Acid	1.29	0.015	higher
2-Hydroxycaproic Acid	1.29	0.015	higher
2-Ethyl-2-Hydroxybutyric Acid	1.12	0.042	higher
Indolelactic Acid	1.11	0.019	lower
Citramalate	1.08	<0.001	higher
Hypoxanthine	1.07	0.003	higher
Urate	1.05	<0.001	lower
Control vs. Pool-II			
Guanine	2.30	0.022	Lower
3-Aminobutyric acid	1.07	0.030	higher

## Data Availability

The data presented in this study are available on request from the corresponding author due to privacy restrictions.

## Supplementary Materials

The following supporting information can be downloaded at: https://www.mdpi.com/article/10.3390/microorganisms14081814/s1, Table S1: Relative abundances of phylum-level microbiota across groups at different time points; Table S2: Relative abundances of genus-level microbiota across groups at different time points; Table S3: Short-Chain Fatty Acid concentrations of in vitro fecal fermentation with different oligosaccharides (mmol/L).
