# Supplementary material for: Effects of Different Types of Pectin Oligosaccharides on the Community Structure and Metabolism of Human Fecal Microbiota
Source: Microorganisms. 2026 Aug 17;14(8):1814. doi: 10.3390/microorganisms14081814 (PMC13515211; doi:10.3390/microorganisms14081814)
Supplement: Supplementary file 1 [file microorganisms-14-01814-s001.zip › microorganisms-4426652-supplementary.pdf]

Table S1 Relative abundances of phylum-level microbiota across groups at different time points

Phylum	Pool-I (12 h)	Pool-I (24 h)	Pool-II (12 h)	Pool-II (24 h)	Positive (12 h)	Positive (24h)	Control (12 h)	Control(24 h)
Firmicutes	50.07 ± 0.88 a	50.55 ± 3.01 a	55.77 ± 3.31 a	48.18 ± 2.13 a	49.17 ± 2.58 a	52.96 ± 0.87 a	56.13± 0.65a	49.03±3.46a
Proteobacteria	44.62 ± 0.96 a	42.34 ± 4.32 a	38.20 ± 3.33 a	42.98 ± 2.30 a	40.88 ± 2.54 a	36.01 ± 1.37 a	37.71± 0.48a	43.93±4.66a
Actinobacteria	3.68 ± 1.04 b	2.71 ± 0.42 b	4.28 ± 0.41 b	4.04 ± 0.48 b	8.49 ± 0.36 a	9.31 ± 0.54 a	4.32±0.29b	3.10±0.10b
Bacteroidetes	0.95 ± 0.07 bc	3.46 ± 1.30 ab	0.99 ± 0.06bc	3.95 ± 0.60 a	0.66 ± 0.07 c	0.93 ± 0.16 c	1.08±0.43bc	2.87±1.40abc

Note: Data are presented as mean ± standard deviation (n = 3). Different lowercase letters (a, b, c) within the same row indicate significant differences between groups according to Tukey's HSD post-hoc test ($p < 0.05$). Two-way ANOVA revealed significant effects of samples (Firmicutes, $p = 0.599$; Proteobacteria, $p = 0.480$; Actinobacteria, $p < 0.001$; Bacteroidetes, p=0.521) and time (Firmicutes, $p = 0.605$; Proteobacteria, $p = 0.566$; Actinobacteria, $p = 0.678$; Bacteroidetes, p=0.013). substrate × time interaction (Firmicutes, $p = 0.013$; Proteobacteria, $p = 0.218$; Actinobacteria, $p = 0.364$; Bacteroidetes, p=0.735).

Table S2 Relative abundances of genus-level microbiota across groups at different time points

Genus	Pool-I (12 h)	Pool-I (24 h)	Pool-II (12 h)	Pool-II (24 h)	Positive (12 h)	Positive (24 h)	Control (12 h)	Control (24 h)
Escherichia-Shigella	21.17 ± 0.50a	19.91±2.53abc	17.78±1.02abc	20.28±0.54ab	19.34±2.22abc	16.58 ± 0.46bc	15.62 ± 0.62c	16.34 ± 0.47bc
Vagococcus	18.12 ± 2.97a	18.43 ± 2.79a	17.16 ± 0.66a	16.45 ± 1.12a	16.34 ± 0.92a	18.72 ± 1.70a	16.43 ± 1.50a	17.55 ± 0.61a
Streptococcus	16.32±2.16ab	12.10 ± 2.13c	16.29 ± 0.66ab	12.71±0.37bc	16.62 ± 1.28ab	18.23 ± 0.22a	13.77 ±0.41bc	11.75 ± 0.32c
Klebsiella	7.65 ± 0.89a	7.41 ± 0.82a	6.41 ± 0.09ab	6.70 ± 0.30ab	5.58 ± 0.42b	5.41 ± 0.28b	5.15 ± 0.32b	5.64 ± 0.05b
Citrobacter	3.60 ± 0.16b	3.66 ± 0.37b	3.40 ± 0.16b	4.22 ± 0.11b	4.20 ± 0.23b	3.76 ± 0.37b	6.07 ± 0.33a	6.93 ± 0.96a
Bifidobacterium	2.58 ± 0.91b	1.83 ± 0.39b	3.18 ± 0.42b	3.27 ± 0.52b	7.61 ± 0.32a	8.38 ± 0.49a	2.98 ± 0.17b	2.41 ± 0.04b
Megamonas	2.60 ± 0.03a	1.73 ± 0.12bc	2.26 ± 0.22ab	1.26 ± 0.35c	2.53 ± 0.22a	1.69 ± 0.22bc	2.27 ± 0.24ab	1.34 ± 0.27c
Phascolarctobacterium	1.00 ± 0.60b	2.69 ± 0.13ab	2.30 ± 0.52ab	2.51 ± 0.46ab	2.20 ± 0.20ab	2.69 ± 0.19ab	3.89 ± 0.24a	3.39 ± 1.04a
Paraclostridium	0.57 ± 0.21bc	0.06 ± 0.03c	6.36 ± 3.98ab	0.71 ± 0.31bc	1.58 ± 0.99bc	0.30 ± 0.13c	9.18 ± 2.36a	0.73 ± 0.09bc
Veillonella	0.09 ± 0.02b	3.92 ± 1.77a	0.16 ± 0.10b	1.71 ± 0.03ab	0.10 ± 0.02b	1.87 ± 0.81ab	0.10 ± 0.05b	3.51 ± 0.95a
Coprococcus_3	0.33 ±0.07b	1.93 ±0.05a	0.34 ±0.03a	1.49 ±0.32b	0.30±0.04b	0.39±0.08b	0.31 ±0.07b	0.33 ±0.06b
Butyricicoccus	0.04±0.01b	1.34±0.43a	0.04±0.01b	0.74±0.03ab	0.03±0.01b	0.69±0.18ab	0.05±0.01b	0.48±0.01b

Note: Data are presented as mean ± standard deviation (n = 3). Different lowercase letters (a, b, c) within the same row indicate significant differences between groups according to Tukey's HSD post-hoc test ($p < 0.05$). Two-way ANOVA revealed significant effects of samples (*Escherichia-Shigella*, p = 0.001; *Vagococcus*, p = 0.806 ; *Streptococcus*, p = 0.003; *Klebsiella*, p < 0.001; *Citrobacter*, p < 0.001; *Bifidobacterium*, p < 0.001; *Megamonas*, p = 0.065; *Phascolarctobacterium*, p = 0.005; *Paraclostridium*, p = 0.013; *Veillonella*, p = 0.192; *Coprococcus_3*, p=0.056; *Butyricicoccus*, p = 0.488) and time (*Escherichia-Shigella*, p = 0.450; *Vagococcus*, p =

0.395; *Streptococcus*, $p < 0.093$; *Klebsiella*, p=0.982; *Citrobacter*, $p = 0.549$; *Bifidobacterium*, $p = 0.729$; *Megamonas*, $p < 0.001$; *Phascolarctobacterium*, $p = 0.158$; *Paraclostridium*, $p = 0.003$; *Veillonella*, $p < 0.001$; *Coprococcus_3*, $p = 0.006$; *Butyricicoccus*, $p < 0.001$). substrate × time interaction (*Escherichia-Shigella*, $p = 0.045$; *Vagococcus*, $p = 0.700$; *Streptococcus*, $p < 0.051$; *Klebsiella*, p=0.775; *Citrobacter*, $p = 0.244$; *Bifidobacterium*, $p = 0.245$; *Megamonas*, $p = 0.969$; *Phascolarctobacterium*, p=0.117; *Paraclostridium*, $p = 0.038$; *Veillonella*, $p = 0.173$; *Coprococcus_3*, p=0.057; *Butyricicoccus*, $p = 0.502$).

Table S3 Short-Chain Fatty Acid concentrations of in vitro fecal fermentation with different oligosaccharides（mmol/L）

Samples	Acetic acid	Propionic acid	Butyric acid
Positive-12 h	20.43±2.32ab	2.28±0.13b	0.24±0.01b
Pool-I-12 h	14.59±0.41a	1.44±0.05a	0.17±0.01a
Pool-II-12 h	14.80±0.99c	1.24±0.65c	0.18±0.01b
Positive-24 h	24.07±0.25ab	4.13±0.52b	1.06±0.28b
Pool-I-24 h	20.27±0.28bc	2.46±0.30c	0.38±0.13b
Pool-II-24 h	20.53±0.76abc	2.43±0.13b	0.38±0.04b

Data are presented as mean ± standard deviation (n = 3). Different lowercase letters (a, b, c) within the same row indicate significant differences between groups according to Tukey's HSD post-hoc test ($p < 0.05$). Two-way ANOVA revealed significant effects of samples (acetic acid, $p < 0.001$; propionic acid, $p = 0.023$; butyric acid, $p = 0.004$) and time (acetic acid, $p = 0.001$; propionic acid, $p = 0.005$; butyric acid, $p = 0.001$). The substrate × time interaction was significant for butyric acid ($p= 0.004$) but not for acetic acid ($p = 0.561$) or propionic acid ($p = 0.211$).
